# Experimental Researches

**Published:** 1851-09

**Authors:** 


### Part 2—Reviews and Notices of New Works.

ARTICLE I.

*Experimental Researches, Illustrative of the Functional Oneness, Unity, and Diffusion, of Nervous Action; in opposition to the Anatomical Assumption, of Four Sets of Nerves, and a Fourfold Set of Functions, and Transmitted Impressions; with a brief Exposition of the Philosophy of Vivisection, and of Sensation.* By BENNET DOWLER, M. D., of New Orleans, &c.

We have, through the politeness of the author, received a pamphlet with the above title, reprinted from the N. O. Med. and Surg. Journal.

Dr. Dowler is one of the few men in our country who have the temerity to doubt the correctness of the theories of "the most distinguished authors," and to publish the results of his own observations when they lead to conclusions at variance with those theories. He, too, is one of the few men in our profession who have the application and ability combined, to prosecute extensive and important original investigations. We know of no one in our country who has been more indefatigable in his labors, or who has developed more striking results. His observations upon post mortem heat, post mortem circulation, previous papers upon the Nervous System, with others of importance and interest, had already gained for him a distinguished position, and the paper before us can but add another sprigg to his laural wreath.

As is generally the case with original obseivers and thinkers, Dr. Dowler has come in for a full share of the animadversion of the critics. He has been sneered at for being remarkable for striking discoveries, and his language has been censured for its peculiarities of style, and strong expressions.

But who has a right to condemn facts for their novelty? especially when developed in a new field of observation, as many of Dr. Dowler have been. And who can express strong ideas and forcible arguments without the use of correspondingly strong language?

To give a correct idea of the paper before us, it would be necessary to reprint the whole article, but this we are sorry our limits forbid us to do. We must, therefore, be content with a mere notice of some of the important conclusions to which the author arrives.

In a former paper, Dr. Dowler reported a series of experiments upon Alligators, by which he arrived at the conclusion that the different parts of the animal were endowed with their due proportion of the sensorium, which seemed to be satisfactorily proved by the fact that the decapitated body moved intelligently in the removal of irritating bodies when applied, as fire, &c., directing its feet so as to remove them, and when unable to succeed it would roll over always from the irritant. In the paper before us the same position is again demonstrated, and the diffusion of the sensorium apparently redemonstrated by experiments upon the same animal.

In addition to this demonstration, by dividing the nerves of the extremeties so as completely to interrupt the connection with the spinal cord, hours after the decapitation of the animal, the Doctor found that muscular motions were excited by compression of the nerve upon the distal side of the point of division; but no impression seemed to be made by compressing it on the other side of the division. From this and other facts, it is inferred that the peripheral portion of the nervous system is the more important, and acts, to some extent at least, independent of connection with the centres. By tracing the nerves toward the extremeties, he found that the nearer he approached to the point of ultimate distribution the more active were the muscular twitchings and contractions, that were excited by their compression.

The tail of the animal after the division of the spinal cord at the point corresponding to the sacral region in man, and the passage of a probe down the caudal canal without any effect, manifested sensation and inteiligential motion as evinced by its moving. "If a piece of ignited paper approached its right side it swayed itself to the left, and so of the contrary." In the two animals vivisected, one was decapitated at first, and the other the brain and spinal cord were destroyed with like general results. The tenacity of life in reptiles makes them better adapted to such experiments as have been performed by Dr. Dowler, than any other class of animals, and to this circumstance he owes much of the success of his experiments.

His conclusions that the sensorium is diffused, and that the nerves are more than mere conductors, seem to be strongly supported by his experiments and arguments; but the part of his paper arguing the functional oneness of the nervous system, we think based upon a more doubtful foundation. His quotations from Magendie to prove that the anterior and posterior roots of the spinal nerves are similar in function, should have been accompanied by an account of the equally recent, and, to say the least, fully as reliable experiments of M. Longet, who, while acknowledging the great difficulty of the experiments, (but great difficulty does not constitute impossibility,) satisfactorily demonstrated to a committee of the Academy of Medicine, and to his classes again and again, that compression of the anterior roots of the spinal nerves of the dog produced motion but no sensation, while a slight touch of the posterior roots invariably caused the animal to yell from pain. This, with the experiments of Sir Chas. Bell, and others, should at least offset the statements of M. Magendie in reference to the results of his experiments, until they shall be confirmed by others. And if there is no perceptible anatomical difference in the structure of nerves, the fact does not prove the unity of their functions against such evidence as that demonstrated to the Academy and to his classes by M. Longet, in corroboration of the doctrines of Sir Charles Bell. But it must be confessed, that it is remarkably difficult to prove conclusively a doctrine that has obtained such universal sanction that one almost stares to hear it doubted; and if Dr. Dowler shall not be able to prove the functional oneness of the whole nervous system, he may do good in setting us to work at hunting up the reasons why we believe that the nerves of sense are peculiar, as the optic, olfactory, &c. are thought to be, and why we believe some nerves are for motion and some for sensation exclusively.

The remarks upon the philosophy of Vivisection are judicious and well timed.

The author seems to be somewhat fearful of the critics, and indulges in what we hope to be an unnecessary foreboding of neglect, and somewhat like the boy in the dark, whistling to keep up his courage, he concludes his article as follows:

"Unlike a prisoner at the bar, an innovator has this consolation, namely, that if the present jury shall condemn him, unjustly, he can look with confidence to a future one, which soon, or late, will do him, and itself, justice, by embracing the truth; although, probably, for a time,

"It will be found upon examination,
That Satan has the largest congregation."

"A hundred cities claimed a Homer dead,
Through which a living Homer begged his bread."

An elegant writer, however, takes a view somewhat different: 'An author,' says D'Israeli, 'must consider himself as an arrow shot into the world; his impulse must be stronger than the current of air that carries him on—else he falls.' The plenitude of an author's complaints must appear, to such as will not accept his contributions, as the veriest of all platitudes. A medical critic, in a foreign Review, says, upon this topic, 'that it is the invariable rule, to invoke the over-worked shades of Harvey and Galileo as illustrations.'"

